# Regeneration of Cone Photoreceptors when Cell Ablation Is Primarily Restricted to a Particular Cone Subtype

**DOI:** 10.1371/journal.pone.0055410

**Published:** 2013-01-30

**Authors:** Brittany Fraser, Michèle G. DuVal, Hao Wang, W. Ted Allison

**Affiliations:** 1 Department of Biological Sciences, University of Alberta, Edmonton, Alberta, Canada; 2 Department of Medical Genetics, University of Alberta, Edmonton, Alberta, Canada; 3 Center for Prions & Protein Folding Disease, University of Alberta, Edmonton, Alberta, Canada; Center for Regenerative Therapies Dresden, Germany

## Abstract

We sought to characterize the regenerated cells, if any, when photoreceptor ablation was mostly limited to a particular cone subtype. This allowed us to uniquely assess whether the remaining cells influence specification of regenerating photoreceptors. The ability to replace lost photoreceptors *via* stem cell therapy holds promise for treating many retinal degenerative diseases. Zebrafish are potent for modelling this because they have robust regenerative capacity emanating from endogenous stem cells, and abundant cone photoreceptors including multiple spectral subtypes similar to human fovea. We ablated the homolog of the human S-cones, the ultraviolet-sensitive (UV) cones, and tested the hypothesis that the photoreceptors regenerating in their place take on identities matching those expected from normal cone mosaic development. We created transgenic fish wherein UV cones can be ablated by addition of a prodrug. Thus photoreceptors developed normally and only the UV cones expressed nitroreductase; the latter converts the prodrug metronidazole to a cell-autonomous neurotoxin. A significant increase in proliferation of progenitor cell populations (p<0.01) was observed when cell ablation was primarily limited to UV cones. In control fish, we found that BrdU primarily incorporated into rod photoreceptors, as expected. However the majority of regenerating photoreceptors became cones when retinal cell ablation was predominantly restricted to UV cones: a 2-fold increase in the relative abundance of cones (p = 0.008) was mirrored by a 35% decrease in rods. By primarily ablating only a single photoreceptor type, we show that the subsequent regeneration is biased towards restoring the cognate photoreceptor type. We discuss the hypothesis that, after cone death, the microenvironment formed by the remaining retinal cells may be influential in determining the identity of regenerating photoreceptors, though other interpretations are plausible. Our novel animal model provides control of ablation that will assist in identifying mechanisms required to replace cone photoreceptors clinically to restore daytime vision.

## Introduction

The adult fish retina possesses a robust innate capacity to regenerate neurons from retinal stem cells [1]–[3], making it an attractive model for stem cell therapies of retinal degenerations. The intrinsic ability to replace cone photoreceptors in fish has been studied following various cell ablation methods, including inflicting retinal neuronal damage from a variety of surgical, toxic light, and toxic chemical lesions [2], [4]–[7]. An intriguing alternative is hormonal induction of UV cone loss that parallels normal development in salmonid fish [8], [9]; UV cones are normally lost during an ontogenetic shift associated with these fish migrating to deeper waters [9]–[12]. Perhaps excluding the latter, available retinal cell ablation methods indiscriminately and inconsistently ablate various photoreceptor subtypes (rods and multiple cone subtypes), along with other cells [13]. Impressively, it appears that all of the ablated cell types are typically replaced during regeneration. The complexity of this suite of regenerating cells has been a roadblock to deciphering the biochemical signalling pathways involved in specifying cell fates during the replacement and rewiring of damaged retina [13].

**Figure 1:**
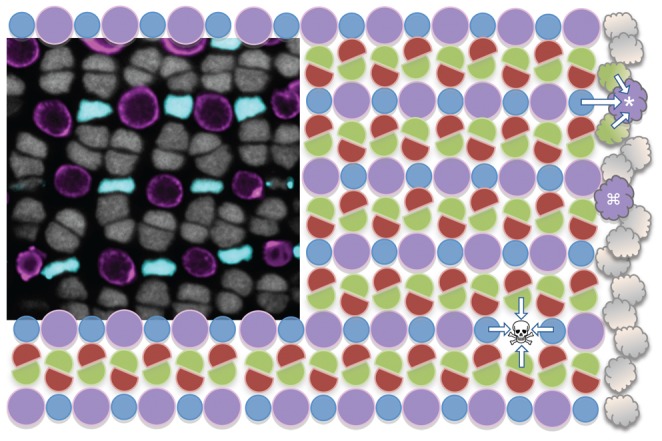
Zebrafish cone photoreceptor mosaic and experimental rationale. The cone photoreceptor mosaic of zebrafish consists of a precise reiterated arrangement of cone spectral sensitivity subtypes, extending in rows radiating toward the periphery of the retina (towards the right). The inset micrograph demonstrates the positions of the UV- and blue-sensitive cones (pseudocoloured magenta and blue, respectively, in a transgenic fish with these cone subtypes expressing GFP and mCherry), and the nuclei of green- and red-sensitive cones forming rows in between (grey). This tangential view of the cones is orthogonal compared to Figure 2, at a level just above the magenta BrdU+ nuclei in panel 2E. This mosaic is schematized, with magenta circles representing the UV cones and other coloured shapes representing their respective cone sensitivities. New cones are continuously added to the periphery of the adult retina from stem cells near the iris (cloud shapes on right); thus the existing mosaic serves as a template for specifying cone identity and/or position of newly added retina. Towards the top right one can imagine a UV cone (*) during differentiation, and its specification being influenced by the identity of the existing mosaic (arrows). Alternatively, mathematical modelling also supports that newly generated cones could be specified without regard for position (e.g. differentiating UV cone marked with ⌘ adjacent to an existing UV cone, this arrangement is not observed in mature mosaic) and subsequently move to their correct position. Our intervention principally ablates UV cones (skull & cross-bones, ☠) and hypothesizes that the fate of the regenerating cell will be influenced (arrows) by the remaining cones.

The signals that specify the identity of photoreceptors during regeneration likely include extrinsic signals from various sources [14]–[18], including from neighbouring cells. Indeed attempts to drive retinal precursors to a cone fate in mouse retinal degeneration models have met with only modest success [19]–[23], despite encouraging progress in replacing rods to restore function [19], [24]–[27], and this is likely due in part to the intrinsically low abundance and density of cones in the murine retina. Thus the cellular environment and neighbour-relationships of photoreceptors are thought to impinge upon the specification of regenerating retinal cells. The abundance of cones in the fish retina is akin to the density of cones in the human fovea, and thus the cone-rich zebrafish retina is advantageous for studies of how stem cell therapies can replace lost cones and restore cone-driven daytime vision in humans. Overall there is deep conservation of photoreceptor structure, function and development from fish to mammals, though a unique feature in the retina of teleost fish is that the cone photoreceptor subtypes are arranged in a precise, reiterated mosaic pattern [28]–[31]. This is well-represented in the adult zebrafish mosaic, which is composed of parallel rows of alternating UV- and blue-sensitive cones, that are adjacent to rows of red- and green-sensitive double-cones (Fig. 1) [28], [29], [31].

This quality of cell arrangements can be referred to as a ‘heterotypic cell mosaic’, wherein cells of different types are spatially arranged in precise patterns relative to one another (contrasting *homo*typic mosaics, wherein cells of a *single* type are spaced in a statistically non-random fashion). Heterotypic cell mosaics are rare (or at least they are rarely easy to recognize) [32] but disparate examples support the contention that paracrine signals from neighbouring neurons can influence cell identity [29], [33]–[35]. This heterotypic cell arrangement, along with the abundance of cones and the innate robust regenerative capacity, combine to compel the cone mosaic of fish as a useful model to assess if extrinsic signals influence the fate of stem cells as they differentiate to replace lost cones in vertebrates [1], [36], [37].

The existence of extrinsic signals that influence the fate of nearby differentiating cones is also supported by inferences drawn from continued growth of the adult fish retina. New photoreceptors are added at the retinal margin throughout the life of fish [38], [39], leading to rings of new retina being added at the retinal periphery (i.e. near the iris) on a daily and weekly basis. These new cones take on positions and identities that preserve the integrity of the elaborate mosaic pattern (schematized in Figure 1, note * at top right) [29], [31], [40]–[42]. The rigorously reiterated pattern amongst the newly added cones strongly suggests that the existing mosaic serves as a template and signal source directing the fate and/or position of newly added cells.

We sought to address the fate of regenerating cone photoreceptors as they differentiate into a relatively intact cone photoreceptor mosaic. To accomplish this, we developed a method of ablating cone photoreceptors of a particular spectral subtype (we chose UV-sensitive cones, the homolog of human “blue cones”). The method enables cone ablation and regeneration that is reduced in complexity compared to existing approaches. We speculated that ablation of UV cones would be sufficient to induce retinal stem cell proliferation, leading to the regeneration of new photoreceptors in their place. Subsequent to confirming this, we turned to addressing alternate hypotheses of photoreceptor specification during regeneration. We tested the hypothesis that regenerating photoreceptors would adopt random identities, refuting the hypothesis that neighbouring cells impose specification on regenerating photoreceptors in our paradigm. On the other hand, increases in rod abundance associated with the loss of UV cones during natural retinal development of trout and other salmonid fish [8], [43], [44] led us to speculate that rods might take the place of ablated UV cones in zebrafish. Alternatively, considering the precise cone mosaic in zebrafish, there is an implication that neighbouring cells signal to each other during development (Fig 1, compare arrows surrounding * at top right and surrounding ☠ at bottom right); thus it may be that UV cones would regenerate in place of ablated UV cone photoreceptors. The latter result might be interpreted as support for paracrine signalling, whereas the former would be interpreted as supporting the existence of conserved intrinsic mechanisms explaining the adaptive loss of UV cones during ontogeny of fish moving to deeper waters. Our results document regeneration of cone photoreceptors, at the expense of a reduced rod genesis, when retinal cell ablation is primarily restricted to the UV cone subtype.

## Results

### Characterizing novel transgenic fish and directed cell-specific ablation of UV cone photoreceptors

**Figure 2:**
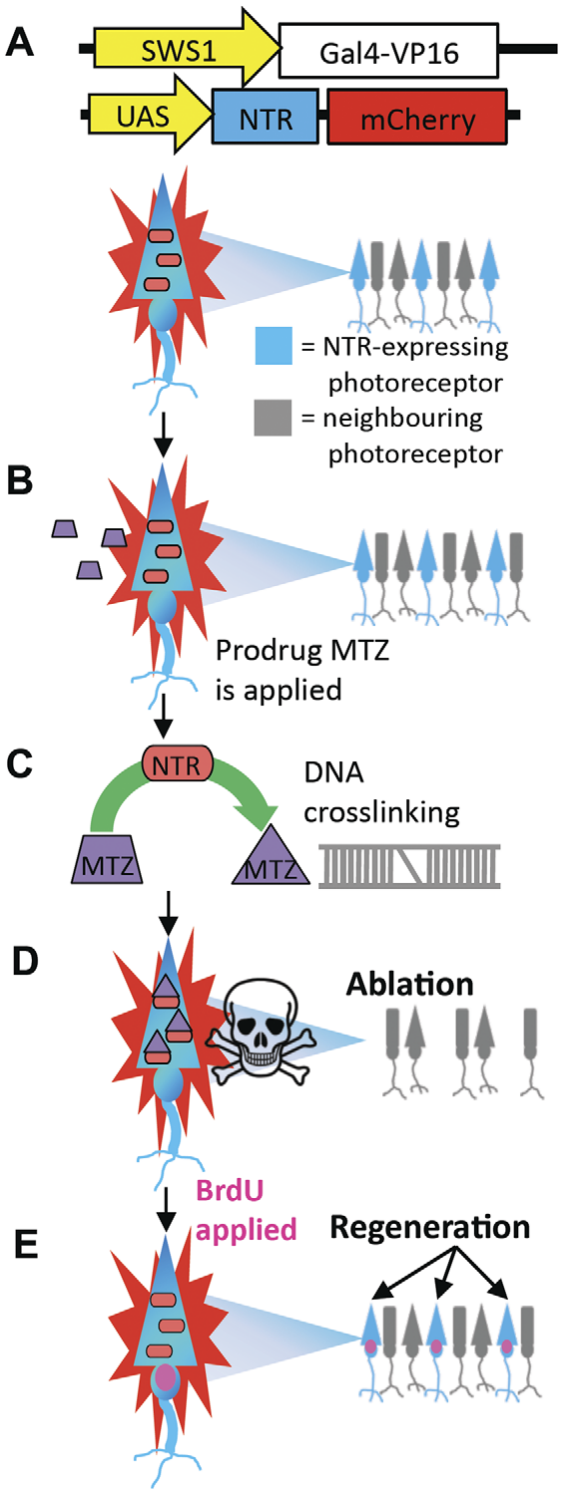
Regeneration investigated via a cell-specific ablation method. We engineered fish where a subset of ultraviolet-sensitive (UV) cone photoreceptors exclusively express the NTR-mCherry transgene (A). Upon treatment with a metronidazole (MTZ) prodrug solution (trapezoid), nitroreductase (nfsb gene, NTR protein) converts MTZ into a cell-autonomous cytotoxin (B, C, represented as a triangle), resulting in DNA crosslinking and ablation of only the cones expressing NTR (D), without disruption to neighbouring cells. Following cell death, BrdU is applied and it incorporates into DNA of proliferating cells (magenta nuclei). Regeneration of the targeted photoreceptors occurs once the MTZ treatment is removed (E).

Transgenic zebrafish were developed wherein UV-sensitive cones could be ablated by addition of a prodrug (Fig. 2). Overall, the goal was to express the bacterial gene nitroreductase (*nfsb* gene encoding NTR protein) in the cones to be ablated; NTR converts the prodrug metronidazole (MTZ) into a cell-autonomous cytotoxin (Fig. 2).

**Figure 3:**
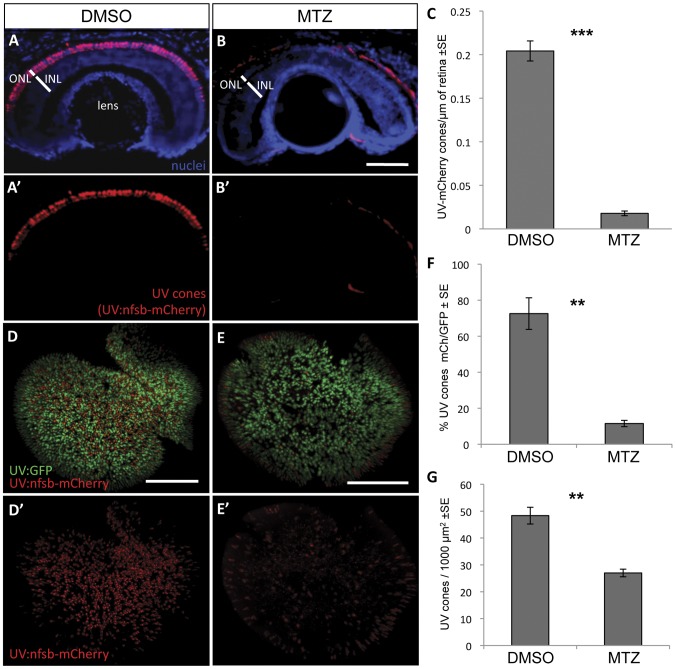
UV cone photoreceptor ablation by prodrug application to transgenic fish. Fish were engineered with inducible cell ablation transgenes expressed in UV cone photoreceptors, *Tg(SWS1:Gal4-VP16)ua3016;Tg(UAS-E1b:NfsB-mCherry)c264* (“UV:nfsb-mCherry”). Fish treated with a vehicle control DMSO solution maintained nitroreductase- (nfsb-) mCherry expression in UV cones and cell death was not induced (A, A’). Siblings of these fish, treated with prodrug metronidazole (MTZ) for 48 hrs, lost the mCherry fluorescence due to ablation of the targeted UV cones (B, B’). Note the red fluorescence in panels B and B’ is auto-fluorescence due to a longer exposure compared to panel A. Quantification of UV cones in these cryosections after treatment with the prodrug MTZ (C) revealed a significant decrease in the number of cones expressing mCherry fluorescence in the ONL compared to vehicle-treated controls (***p<0.0001; DMSO treated n = 10, MTZ treated n = 8). Similar observations were made on flat-mounted retina (D,E) wherein the UV cones expressing nfsb-mCherry decreased in abundance relative to the number of UV cones expressing GFP (F, **p<0.001; n = 9 DMSO-treated, n = 10 MTZ-treated) or when considering the absolute density of all UV cones per unit area (G, **p<0.001). Scale bar  =  50 µm in A,B and 100 µm in D,E.

Expression of NTR-mCherry fusion protein was characterized, based on mCherry fluorescence in sectioned and wholemount retinas, in transgenic zebrafish with Gal4-VP16 in UV cones, driving expression of NTR-mCherry, *Tg(SWS1:Gal4-VP16)ua3016;Tg(UAS-E1b:NfsB-mCherry)c264*. The transgenic fish line *Tg(SWS1:Gal4-VP16)ua3016* is a novel line intended to express Gal4-VP16 in UV cones; we first asked if the expression of this transgene was limited to UV cones and was consistently present in all UV cones, as we expected. This line was crossed to *Tg(UAS-E1b:NfsB-mCherry)c264* [45] and mCherry expression was observed in the photoreceptors of fish expressing Gal4-VP16 (confirmed by expression of GFP in the heart, as engineered into the transgene constructs). Overall, the mCherry was contained exclusively in UV cones (Fig. 3A,B), though not in all UV cones, as revealed in *Tg(SWS1:Gal4-VP16)ua3016;Tg(UAS-E1b:NfsB-mCherry)c264;Tg(-5.5opn1sw1:EGFP)kj9* fish (Fig. 3D). We also noted an apparent decrease in the number of cones expressing mCherry with age, precluding utility of this line for the study of cone ablation in adult fish (Fig. S1). This is consistent with recent reports indicating that the repetitive 14XUAS sequence we used becomes disabled during development due to gene silencing [46]–[48]. However we also note that crossing the above lines to an independent 4XUAS line (creating *Tg(SWS1:Gal4-VP16)ua3016;Tg(UAS-E1b:NfsB-mCherry)c264*;*Tg(4xUAS:GFP)hzm3*) suggested that Gal4 is not expressed in all UV cones (Fig. S1), so this may also be a limitation imposed by our *Tg(SWS1:Gal4-VP16)ua3016* driver line.

The quality and quantity of UV cone ablation following prodrug application was assessed. Following MTZ treatment for 48 hours, *Tg(SWS1:Gal4-VP16)ua3016;Tg(UAS-E1b:NfsB-mCherry)c264* zebrafish larvae showed an obvious reduction in mCherry fluorescence within the photoreceptor layer of the retina (Fig. 3A,B). Sections of individual fish retinas were examined to quantify the reduction of NTR-mCherry expressing UV cones. Cones expressing mCherry per section were decreased in abundance by 91.3% following MTZ application compared to fish that did not receive the prodrug, and this difference was significant (p< 0.0001, control n = 10 fish, experimental n = 8 fish) (Fig. 3C). As an alternative quantification approach, this experiment was repeated in fish with the genetic background *Tg(-5.5opn1sw1:EGFP)kj9* that produce GFP expression in the UV cones [49]. UV cones expressing GFP are more abundant than those expressing nfsb-mCherry, and thus serve as a useful benchmark for quantification and assessing the specificity of our interventions. Retinae were dissected from the larvae and flat-mounted (Fig. 3 D,E) to quantify the number of UV cones (containing GFP) that expressed the transgene and were ablated by MTZ. In fish receiving vehicle control, 72±8.7% of the GFP+ UV cones were mCherry+ (n = 9). Two days following prodrug application, 12±1.7% of the GFP+ UV cones were mCherry+ (n = 10). Thus prodrug application ablated UV cones expressing the transgene with good efficiency, inducing a 6- fold reduction in NTR-mCherry expressing UV cones (Fig. 3F, p<0.001). We also quantified that the density of GFP-positive UV cones per unit area was reduced by approximately half following MTZ application (Fig. 3G), and this accords well with mCherry-positive UV cones (70% of the GFP-positive cones) being ablated with good efficiency reported above.

**Figure 4:**
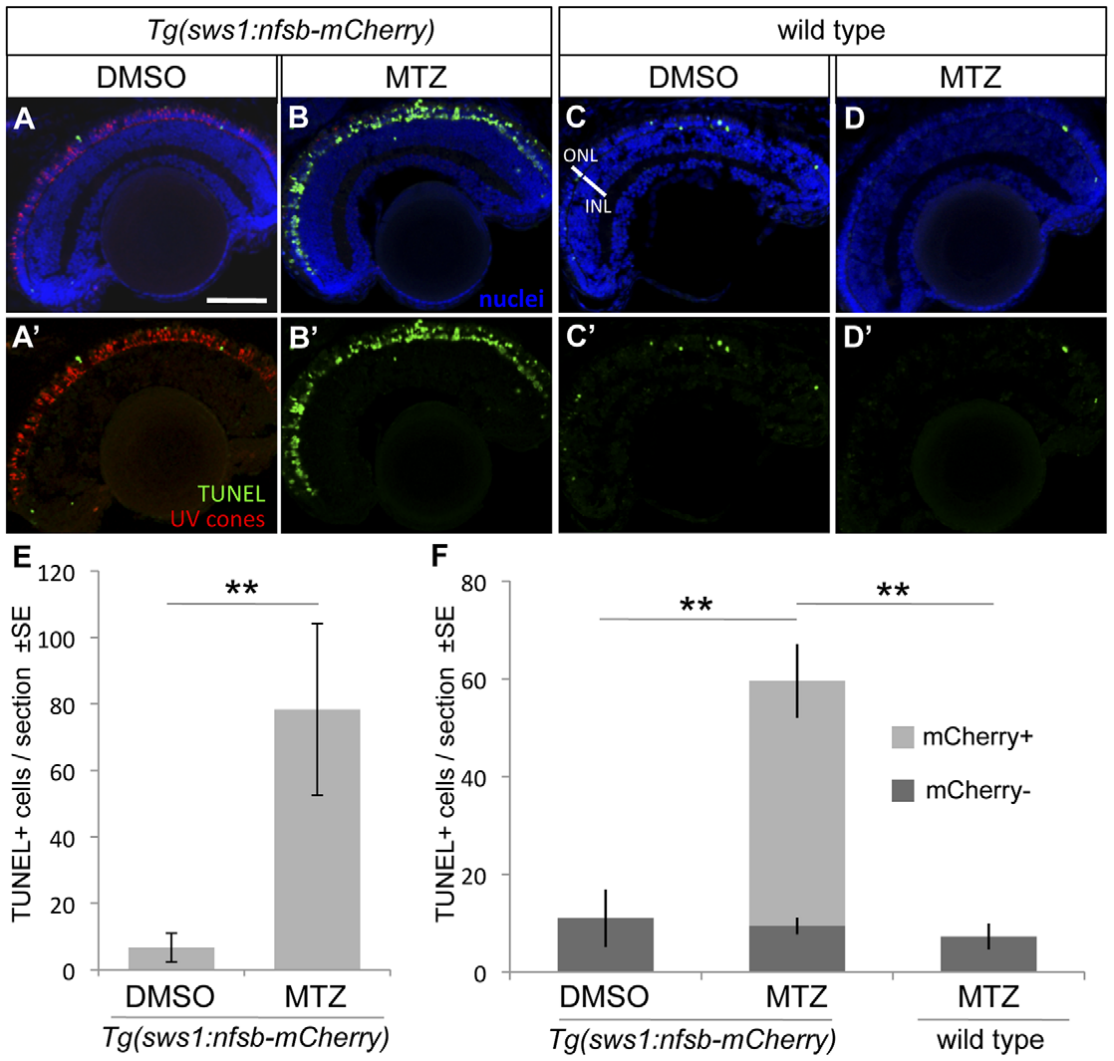
UV cone photoreceptor death induced by prodrug application to transgenic fish. An abundance of apoptotic cells were detected in the ONL of the retina of *Tg(SWS1:Gal4-VP16)ua3016;Tg(UAS-E1b:NfsB-mCherry)c264* fish treated with MTZ for 48 hrs (B, B’) compared to various controls. Very little apoptosis was found in the ONL of *Tg(SWS1:Gal4-VP16)ua3016;Tg(UAS-E1b:NfsB-mCherry)c264* fish treated with a control DMSO solution (A, A’). Non-transgenic siblings treated with either DMSO or MTZ (C or D, respectively) for 48 hrs also possessed few apoptotic cells. **E**. The number of TUNEL+ cells in retinal sections equivalent to A and B were quantified, showing an order of magnitude increase upon MTZ prodrug application (**p<0.001, MTZ-treated n = 11, DMSO treated n = 15). **F**. A repetition of this labelling to discern the bystander effect, i.e. compare the number of TUNEL+ cells that do not express nfsb-mCherry as compared to basal levels in normally developing fish. Again the total number of TUNEL+ cells is increased when the transgenic fish are treated with prodrug MTZ rather than DMSO vehicle control (compare total height of light+dark bars, MTZ-treated n = 7). This contrasts the abundance of TUNEL+ cells without mCherry, which is not increased relative to DMSO control fish nor to wildtype fish receiving MTZ (dark grey bars, p = 0.84 and p = 0.927, respectively wherein n = 4 or 3, with means comparable to panel E). Scale bar  =  50 µm.

Abundant TUNEL staining was observed in cone photoreceptor nuclei after exposure to MTZ (Fig. 4), coincident with the decrease of transgene-expressing UV cones, confirming that the loss of mCherry fluorescence is in fact due to cytotoxin-induced ablation via apoptosis. The abundance of TUNEL-positive cells was an order of magnitude greater in the ONL of fish receiving MTZ compared to those receiving vehicle control (78.3±8.6 vs. 6.7±1.5 TUNEL+ cells per retinal section, ±SE. n = 9 and 8 fish, respectively p<0.001). The cell-specific expression of NTR was at a sufficiently high level to form a viable toxin upon the addition of MTZ, confirming that this germline transmissible technique is valid for temporally inducible targeted-ablation of cone photoreceptors.

To ensure that MTZ application did not kill or damage cells beyond those expressing the nfsb-mCherry transgene, consistent with past work showing lack of bystander effects [50]–[55], we quantified the number of dying cells that were not expressing the nfsb-mCherry transgene. We reasoned that if bystander effects were minimal then the abundance of these cells should not exceed the basal level of retinal cell death in normally developing larvae. We repeated the TUNEL labelling immediately above on an additional set of larvae. In *Tg(SWS1:Gal4-VP16)ua3016;Tg(UAS-E1b:NfsB-mCherry)c264* transgenic larvae receiving MTZ the majority of TUNEL-positive cells expressed mCherry (84±3%, n = 7). To assess if a bystander effect was occurring, we identified cells characterized as being both TUNEL-positive and mCherry-negative, and compared their abundance to the basal abundance of TUNEL-positive cells in control retinas (Fig. 4F). The TUNEL-positive ONL cells that lacked mCherry in the transgenic fish receiving MTZ (9.4±1.7 cells per section) were not more abundant compared to transgenic fish receiving DMSO vehicle (7.3±2.7, p = 0.84) or to wild type fish receiving MTZ (11.0±5.9, p = 0.927). This same conclusion was extended to retinal cells outside the ONL (2.0±0.6 cells in transgenic fish receiving MTZ compared to 5.3±0.3 (p = 0.996) and 4.5±0.9 (p = 0.97), respectively). An increase in total TUNEL-positive cells was again noted in transgenic fish receiving MTZ (59.6±8.2 cells ± SE, Fig. 4F considering the total height of all bars per treatment) compared to transgenic fish receiving DMSO vehicle only (7.3±2.7, p = 0.001) or compared to wild type fish receiving MTZ (11.0±5.9, p = 0.004). In sum, MTZ application did not appear to induce death of cells beyond the targetted nfsb-mCherry cells, because the abundance of off-target dying cells was similar to the basal level of dying cells in normally developing retinas.

**Figure 5:**
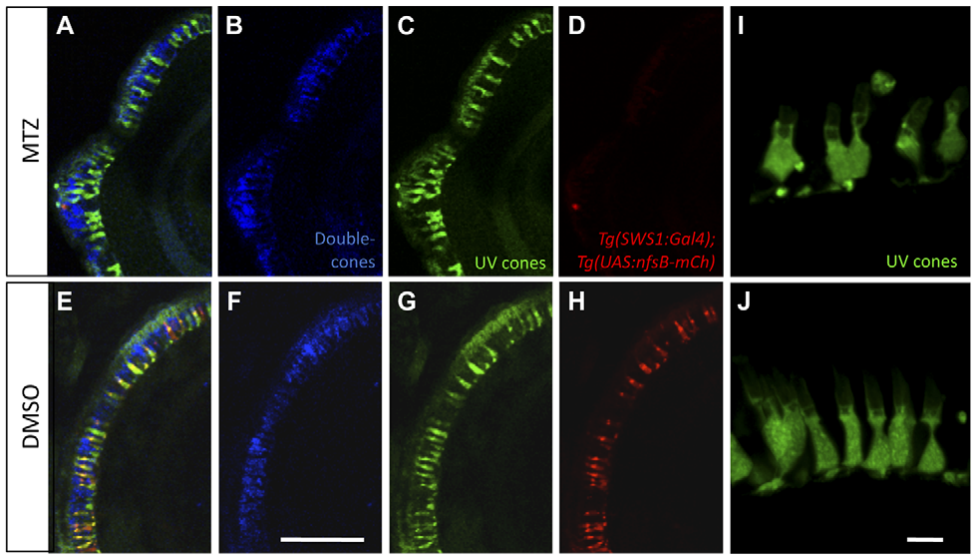
Morphological analysis of photoreceptors indicates that metronidazole does not damage neighbouring cells. UV cones expressing nitroreductase (NTR) were ablated after the addition of prodrug metronidazole (MTZ) (A–D), while *nfsB*-mCherry persisted in transgenic fish treated with DMSO vehicle control (E–H). 24 hours after ablation, UV cones lacking NTR-expression appeared morphologically normal (C) based on their GFP expression compared to controls (G). Red-green double cone pairs were detected with the antibody zpr1. Zpr1 labelling was consistent following MTZ ablation (B) showing normal cell morphology compared to the controls (F). Scale bar  =  25 µm. A magnified view of remaining UV cones (I) with normal morphology comparable to controls is presented from a separate sample (J, Scale bar  =  5 µm).

Because the cell death measurements above can only reveal the potential bystander effects during a brief snapshot in time, we also compared the gross morphology of the remaining cones following prodrug application. Zebrafish *Tg(-5.5opn1sw1:EGFP)kj9* that produce GFP expression in the UV cones [49] were crossed into *Tg(SWS1:Gal4-VP16)ua3016;Tg(UAS-E1b:NfsB-mCherry)c264* such that NTR-mCherry expression was present in a subset of the UV cones and GFP was present in all UV cones. Zpr1, an antibody that labels the red-green double cone photoreceptors, was applied to determine if neighbouring cells were affected by ablation. 24 hours following MTZ treatment, the subset of UV cones expressing NTR were ablated, while neighbouring double-cone pairs and UV cones lacking NTR-expression persisted and appeared morphologically normal (Fig. 5). Examining the remaining UV cones with confocal microscopy further supported this (Fig. 5I, J). Thus neither by examining morphology of neighbouring cells nor by quantifying TUNEL labels were we able to support the hypothesis that adjacent cells were damaged. This is consistent with past work showing that MTZ does not have a toxic bystander effect on neighbouring cells, including neighbouring retinal neurons [50]–[55].

### Effects of cone photoreceptor ablation on retinal stem cell proliferation

We tested whether our combination of transgenes and MTZ prodrug that resulted in UV cone cell death subsequently led to increased proliferation in the retina. Abundance of proliferating cells was assessed in stem cell populations known to occur in the retina via detection of BrdU incorporation.

**Figure 6:**
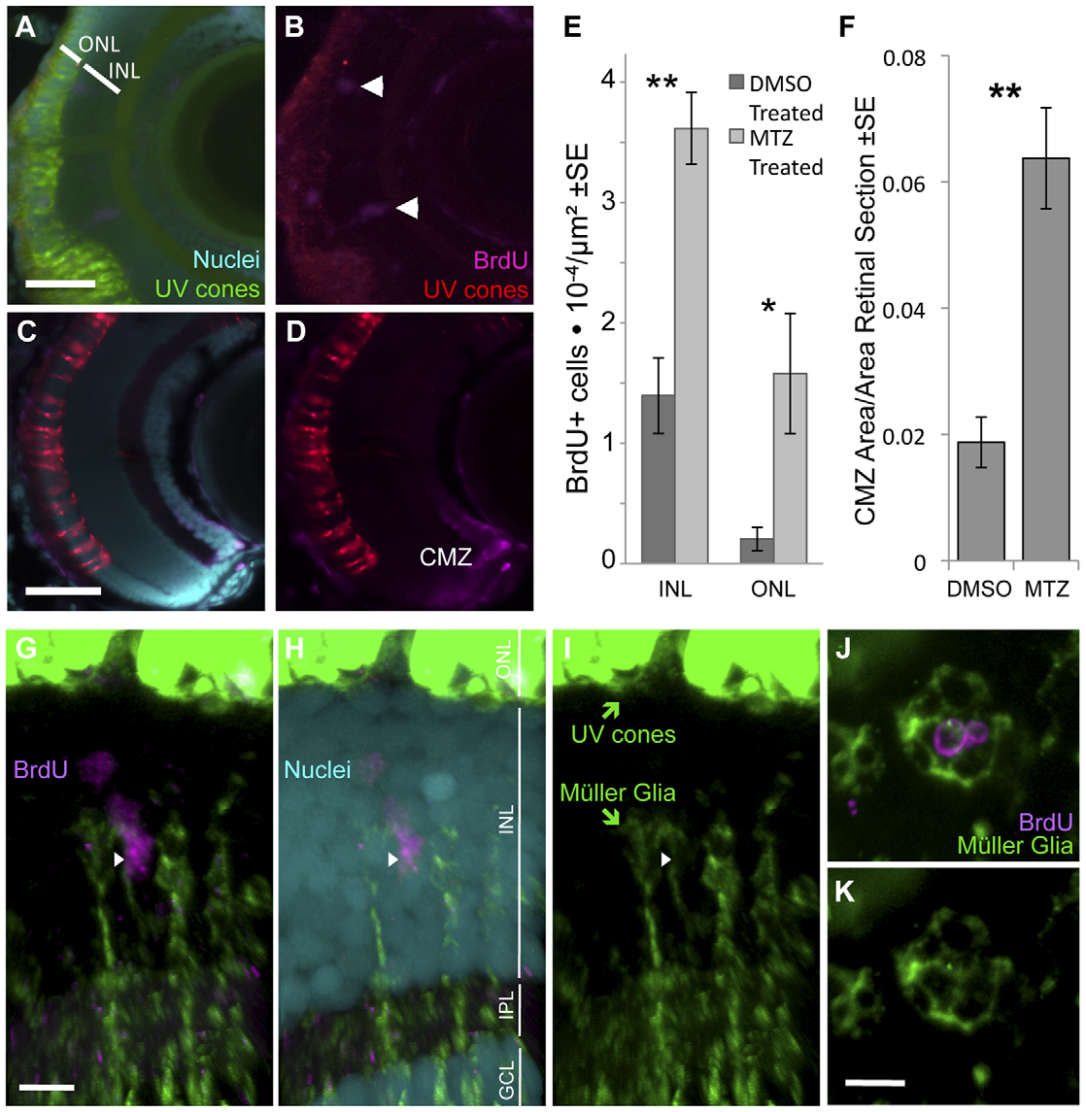
Ablation of cones, primarily UV cones, is sufficient to induce an increase in progenitor cell proliferation. Our transgenic UV cone ablation model fish treated with prodrug MTZ showed a significant increase in proliferating cells in the retina at 24 hours after ablation (A, B, especially notice cells demarcated with arrowheads in top right away, from the retinal margin (ciliary marginal zone, CMZ)) compared to sibling fish treated with a DMSO vehicle control solution (C, D). BrdU is incorporated into the CMZ of all fish with and without cone ablation, as expected (A–D). Proliferating cells were quantified in the inner and outer nuclear layers (INL and ONL) at 24 hours after ablation (E) (**INL p = 0.004, *ONL p = 0.002, DMSO treated n = 9, MTZ treated n = 10). The area of the CMZ was also greater in extent after MTZ treatment, calculated relative to the area of the entire retinal sections (F, **p<0.001, MTZ-treated n = 11, DMSO treated n = 15). Scale bars  =  5 & 3 µm in A & C, respectively. **G–I**. The proliferating cells that increase in abundance during regeneration include Müller glia in the INL, as indicated by close apposition of Müller glia markers (green) with BrdU+ nuclei (magenta) (arrowhead). Example shown is from Movie S1 with a cocktail of two antibodies against Müller glia (green, anti-GFAP & anti-glutamine synthetase, both raised in mouse. Saturated green at top of figure is from UV cones expressing abundant GFP, scale bar  =  5 µm) and anti-glutamine synthetase antibody produced similar results alone (Movie S2). ONL, outer nuclear layer; INL, inner nuclear layer; IPL, inner plexiform layer; GCL, ganglion cell layer. **J–K**. Same labelling regimes as panel G but as a tangential section (orthogonal to G and equivalent plane to Figure 1), showing BrdU+ nuclei in the INL surrounded by Müller glia. Scale bar  =  5 µm.

Observations of retinal sections showed the expected incorporation of BrdU into the ciliary marginal zone (CMZ) of all transgenic fish, both with and without MTZ treatment, indicating that BrdU successfully integrated into the newly synthesized DNA of proliferating retinal neurons at the time of exposure [56]. An increase in proliferating cells was quantified following MTZ ablation in the known populations of proliferating cells in the mature portions of the retina (excluding the CMZ). At 24 hours following BrdU application, a significantly greater number of BrdU-positive cells were present in both the inner and outer nuclear layer (INL and ONL) in fish receiving MTZ compared to those that received vehicle control (Fig. 6A–D). 24 hours after ablation, fish treated with the prodrug showed an approximate 2.5-fold increase in BrdU labels in the INL (p<0.004, control n = 9, experimental n = 10) and an 8-fold increase in the ONL (p = 0.002) of the retina (Fig. 6E). The long pulse of BrdU used in these experiments may have allowed time for cells to migrate between compartments. The area occupied by the CMZ was also quantified, as this developing retina near the iris is constitutively proliferative and adds new retina throughout the life of the fish. In addition to increases in ONL & INL progenitor population sizes, the size of the CMZ was larger in fish receiving MTZ compared to those that received vehicle control (Fig. 6F) (0.064±0.008 vs. 0.019±0.004 as a ratio of BrdU-positive-CMZ area to total retinal area ±SE, n = 11 and 15 fish, respectively p<0.001). The increase in proliferation following conditional ablation, principally of the transgenic UV-sensitive cone photoreceptors, provides evidence that the limited neuronal death of a subset of a single cone photoreceptor type was sufficient to trigger an injury response.

Considering that retinal regeneration in adults is accepted to be borne by Müller glia as a major source of stem cells [57], [58], we were especially intrigued by the dramatic increase in BrdU+ cells of the INL where Müller glia reside. We sought to clarify if Müller glia were contributing to the regenerative response by assessing if the INL BrdU+ nuclei were surrounded by Müller glia markers. Immunohistochemistry using established antibodies, anti-GFAP and/or anti-glutamine synthetase, revealed that Müller glia enveloped the BrdU+ nuclei. The antibodies were most effective as a cocktail (both are raised in mouse, Fig. 6G–I and Movie S1) but were also effective when anti-glutamine synthetase was applied alone (Movie S2). Slices through the tangential plane clarified that BrdU+ nuclei were enveloped by Müller glia (Fig. 6J–K, Movie S3). Thus cone ablation, principally limited to ablation of UV cones, is sufficient to induce proliferation associated with Müller glia serving as retinal stem cells.

### Regeneration following conditional ablation of UV cone photoreceptors

**Figure 7:**
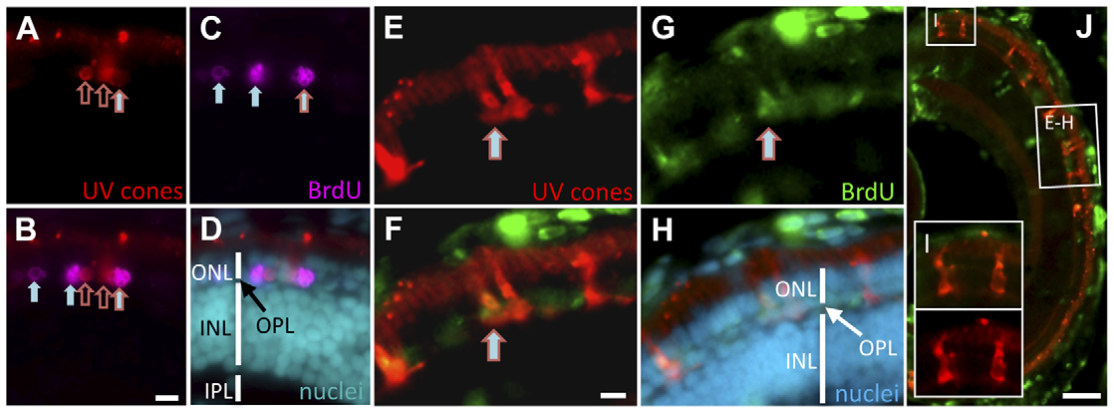
Regeneration of UV cones following prodrug cell ablation. Regenerating UV cones were observed in larvae 5 days after MTZ treatment. Co-localization of these UV cone markers with BrdU detection indicated that the UV cones were recently proliferating. The right-most cell (A–C) is double labelled for BrdU and UV opsin (mCherry) (filled arrow with red outline; has not yet differentiated to a cone morphology). Other UV cones have begun to reappear (empty red arrows), presumably outside of the window of BrdU application. Other BrdU-positive photoreceptors are detectable (filled arrows), likely representing rods, nascent UV cones, or UV cones not expressing the transgene. One week after MTZ ablation, the regeneration of morphologically mature cones was observed (E–H). BrdU+ cells co-labelled with UV cone mCherry expression (E–G; arrow). The regenerated UV cones are morphologically similar to newly generated UV cones in the expanding retinal margin (I), demonstrating that the regenerated cones are qualitatively normal. Scale bars for A–H  =  5 µm, J  =  50 µm.

We tested the hypothesis that cone photoreceptor ablation, principally ablation of a particular cone photoreceptor subtype, is insufficient to induce regeneration of new cone photoreceptors. Larvae were first examined for evidence of regeneration at 5 days post MTZ treatment. BrdU-positive detection of cells in the ONL of the retina indicated that the regenerating retinal neurons were recently proliferating (Fig. 7). Although the regenerating cells were not yet morphologically mature, in this transitional state, the co-localization of BrdU incorporation with the expression of NTR-mCherry indicated that neuronal precursors had begun to differentiate, and had an established cone photoreceptor fate as indicated by the expression of UV opsin transgene (Fig. 7A–D). Other BrdU-positive cells were observed that did not co-localize with mCherry (Fig. 7B). These retinal neurons potentially represent regenerating UV cones not expressing the NTR transgene. It is also possible that these neurons had entirely different cell fates and differentiated into another type of cone or rod photoreceptor, or were constitutively proliferating rod precursor cells.

The regeneration of morphologically mature cones was observed 1 week after MTZ ablation. BrdU-positive cells that co-localized with UV cone NTR-mCherry transgene expression were detected (Fig. 7E–H). Regenerated UV cone photoreceptors were observed to be morphologically similar to UV cones generated during normal development (e.g. compared to Fig. 5 G, H, J) and to cells lacking BrdU in the peripheral expanding retina (Fig. 7E–H, I, perhaps representing newly generated UV cones), suggesting that the regenerated neurons are qualitatively normal. Overall, assuming our treatments did not induce damage to other cells in the retina (consistent with Fig. 4 and 5), these data refute the hypothesis above by demonstrating that ablation of a small set of cone photoreceptors is sufficient to induce regeneration of cone photoreceptors.

### Identity of regenerating cone photoreceptors after the targeted ablation of a subset of UV cone photoreceptors

We speculated that the ablation of a particular cone type would lead to the regeneration of rod and cone photoreceptor subtypes in random abundances (see Introduction). NTR-transgenic larval zebrafish were submitted to a series of treatments between 7 and 14 dpf with MTZ to induce UV cone-specific ablation, and to incorporate BrdU into proliferating cells (24 h BrdU at 7 dpf; 24 h MTZ or DMSO at 8 dpf; 48 h naught at 9–10 dpf; 24 h MTZ or DMSO at 11 dpf; 24 h naught at 12 dpf; 48 h BrdU at 13–14 dpf, see Methods). The larvae were reared for 3–5 months, allowing the eyes to grow large enough to enable retinal dissections, freeing the neural retina from the retinal pigment epithelium (RPE). An examination of the whole-mount neural retinas for BrdU-containing photoreceptors within the larval remnant was then performed.

**Figure 8:**
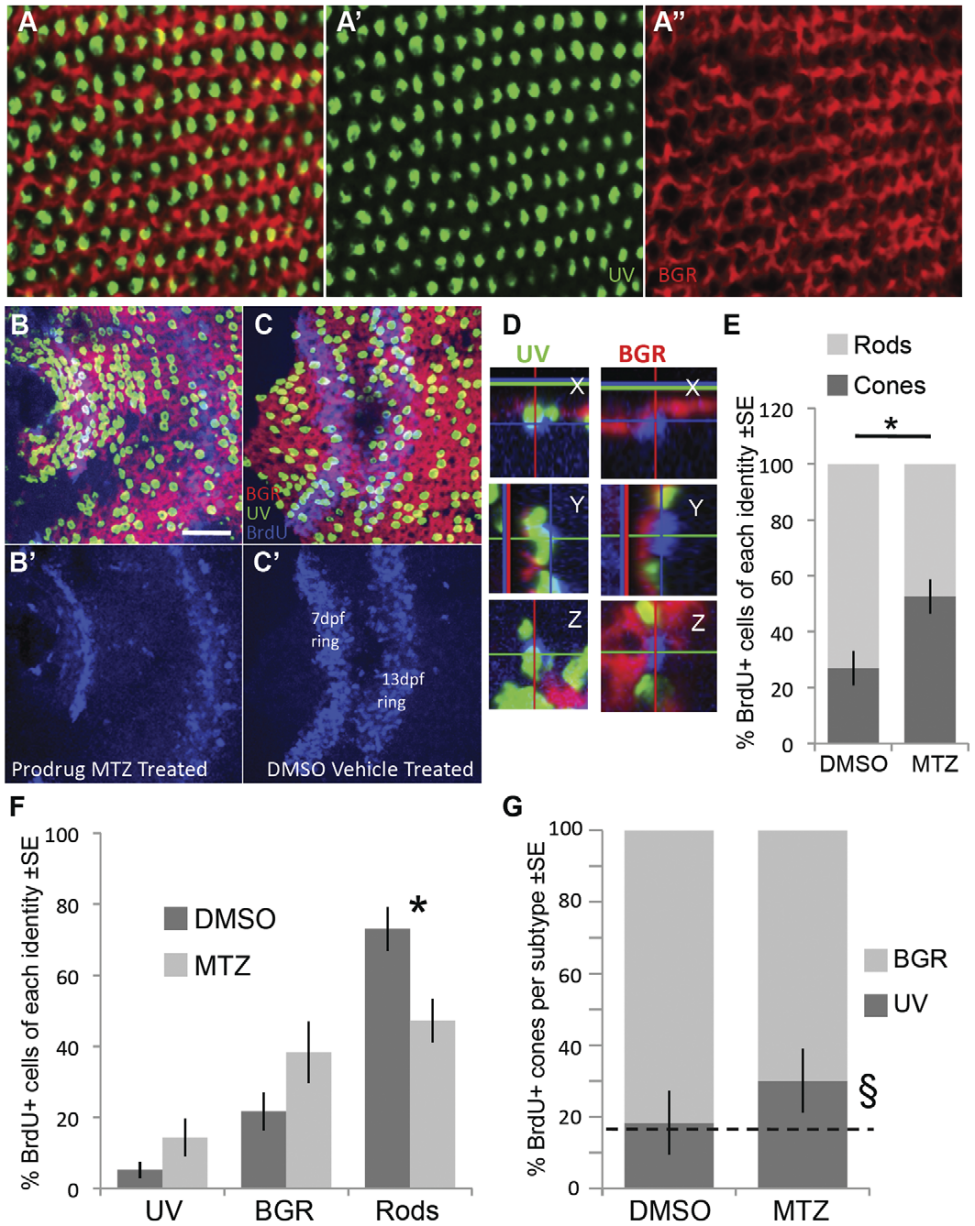
Following ablation of UV cones, the regenerating photoreceptors were more likely to become cones. Opsin riboprobe labelling to quantify the identity of regenerating cones: Throughout this figure, UV opsin riboprobe is green, and a cocktail of blue-, green- & red-opsin riboprobes are labelled in red. **A**. Post-larval areas of adult retina have the expected row mosaic pattern (see Figure 1) A’ & A’’ present individual riboprobes merged in A. **B,C**. Retinas labelled and oriented as per panel A (see also Figure 1), with BrdU located near the less-well-organized optic nerve head. Two rings of BrdU are visible marking cells that were dividing at the retinal periphery during BrdU application (7 & 13 days post fertilization, dpf, also shown separately in B’,C’). Only BrdU+ cells located between the two rings or inner to the 7 dpf ring were analyzed for co-localization with a photoreceptor subtype marker. Panel B is retina that had UV cones ablated by application of prodrug, panel C is control retina receiving vehicle control only. **D**. Magnified view of BrdU+ cells (blue) in three dimensions from images equivalent to panel B; images in panel B are presented in the ‘Z view’. Cell in the left column was categorized as a UV cone because the UV opsin riboprobe label (green) is contiguous with the apical side of the BrdU+ nucleus. Cell in the right column was classified as a BGR (Blue, Green or Red cone) because the cocktail of these opsin riboprobes labels (red fluorescence) an area contiguous with the apical side of the BrdU+ nucleus. Apical is up in the X view, and to the left in Y view. **E**. As expected, most BrdU+ cells in control retina are rods, contrasting results following UV cone ablation by MTZ application, where the majority of BrdU cells are cones (* p = 0.008 by Kruskal-Wallis analysis of variance, N = 6 fish each in MTZ and DMSO vehicle control, equally divided amongst two trials). **F**. The number of BrdU+ cells per photoreceptor subtype (sub-dividing the results in panel E). Normal control retinas mostly generated rods, and the proportion of cone subtypes is coordinately but non-significantly increased. **G**. The relative abundance of BrdU+ UV cones, compared to other BrdU+ cone subtypes, did not increase significantly (p = 0.064) as assessed by a non-parametric analysis of variance per fish (N = 6). The proportion of UV cones matched the expected frequency of 16.7% (dotted line) in DMSO vehicle control treated fish (18.3%, χ^2^ p = 0.769, n = 84 BrdU+ cones), but was higher than the expected frequency following UV cone ablation when assessed as a sum of all fish (§, 30.1%, χ^2^ p = 0.0002, n = 311 BrdU+ cones in 6 fish). Overall, cone genesis increased following cone ablation, and future work is needed to determine the extent to which UV cones are more likely to regenerate relative to other subtypes following UV cone ablation.

Among the twelve fish retinas examined (control n = 6, experimental n = 6), the photoreceptor pattern in the larval remnant in the adult retina was observed to be variable, consistent with past data [31]. Similar to the adult zebrafish retina, the larval retina displays a mosaic pattern; however it is not as strictly defined as the intricate adult cone mosaic (Fig. 8A–C) [31]. Specifically, the precise rows of cone photoreceptors do not form until later in development. Photoreceptors generated during larval development retain a somewhat disorganized larval pattern (though some remodelling is speculated to occur) making the larval remnant, proximal to the optic nerve, distinctive in its cone spacing [31]. Overall, it was observed that MTZ treated NTR-transgenic retinas contained more BrdU-positive cells in the ONL tough this difference was not significant (average number of BrdU+ cells: MTZ treated = 124.3±29, DMSO treated = 66±11, p = 0.10), showing a similar trend with data in Figure 6, further supporting our hypothesis that UV cone ablation induces an increased level of proliferation.

BrdU-containing cells in the larval remnant were classified into six categories based on opsin *in situ* hybridization, confocal microscopy and nuclear position by an observer blinded to the treatments. We focused on those cells we could unambiguously categorize (see Methods, where we describe that ambiguous identities were rare and not differentially abundant between treatments) and thus consider three categories of regenerated (BrdU-positive) cells: i) UV cones; ii) BGR cones (other cone photoreceptors); and iii) rod photoreceptors. The abundance of each of these was tallied, and the percent contribution of each category to the total BrdU-positive cells was determined.

In retinas that received vehicle only, the majority of cells containing BrdU were rods (mean of six retinas = 73±6%), as expected. BrdU-positive UV cones (5.2±2%) and BGR cones (21.7±5%) were also detected, which was not expected. The appearance of cone photoreceptors in this area can be taken as evidence of remodelling of the larval remnant during maturation, as one of us recently speculated may occur [31].

We next tested the hypothesis that death of UV cones would lead to an increase in the number of rod photoreceptors, as observed in the trout retina [8], [43]. Unexpectedly, the proportion of recently proliferating cells that differentiated into rods (47.3±15%) was reduced by almost half following UV cone ablation, compared to controls without ablation (73±6%; p = 0.008). This observation demonstrates that when UV cones are selectively destroyed, the resulting gaps in the photoreceptor mosaic are not preferentially replaced by rods (Fig. 8E, F). This data rejects the hypothesis that ablation of UV cones would lead to an increase in the number of rod photoreceptors.

Mirroring the decrease in BrdU-positive rods, a two-fold increased proportion of BrdU-positive cells were identified as cones following ablation of UV cones (Fig. 8E, 27±7% for control fish, 53±7% for fish receiving MTZ, p = 0.008). Thus ablation of a cone subtype led to preferential regeneration of cones. We next considered the typical identity of regenerating cones following ablation of UV cones. Amongst BrdU-positive photoreceptors, retinas receiving MTZ had a nearly 3-fold (175%) increase in UV cones (14.4±5% of BrdU-positive cells compared to 5.2±2% in vehicle-treated controls) (Fig. 8F). In addition, MTZ treated retinas had a smaller (77%) increase in BrdU-positive BGR cones (38.3±9%) compared to the control retinas (21.7±5%) (Fig. 8F). This evaluation of cone subtypes as a percentage of BrdU-positive photoreceptors did not rise to statistical significance in our small sample size (n = 6 animals per treatment) using a non-parametric analysis of variance (Figure 8F, p = 0.064 and p = 0.128 for UV and BGR cones respectively, Kruskal-Wallis test).

Intriguingly, UV cones were non-significantly increased in their relative abundance compared to expected values amongst the cone subtypes. The relative abundance of each cone subtype is canonical, because red and green cones occur twice as frequently as UV or blue cones (e.g. see the reiterated mosaic of cones in Figure 1, reviewed in ref [31]), such that UV cones make up one sixth (16.7%) of the cones. Not surprisingly, the control (DMSO vehicle treated) retinas examined herein bear this out, with 18.3% of BrdU-positive cones being UV cones, which is not different from the expected proportion (Fig. 8G, χ^2^ p = 0.769, n = 84). Following ablation of UV cones, 311 BrdU-positive cones were observed and 30.1% of them were UV cones, which is different from expected (Fig. 8G, χ^2^ p = 0.00024) but fails to consider inter-individual variation.

In summary, the data support that non-random photoreceptor regeneration occurs, especially including an increased cone genesis at the expense of rod genesis, when cell ablation is principally limited to UV cones; however future work is required to resolve the disagreement amongst our non-parametric statistical analyses as to whether or not these regenerated cones have any bias to differentiate into UV cones (see Discussion).

## Discussion

### Precise cell ablation facilitates tractable regeneration models

Indeterminate growth in fish includes continued expansion of the retina, and this proliferation underpins robust retinal regenerative capacity. In the normally growing adult fish retina, cells are added at the retinal periphery near the iris, and the retina stretches like an expanding balloon. The latter is especially relevant to regeneration, as Müller-glia-derived stem cells throughout the mature retina generate ‘rod progenitors’ and subsequently new rod photoreceptors are added into the expanding retina [58], [59]. Thus rod density remains somewhat constant despite stretching of the retina during growth [60]. These stem cells are able to regenerate all retinal cell types, including cone photoreceptors, in response to damage [4], [5], [61]–[64].

In contrast to rod photoreceptors, cones are not thought to be added into the mature portions of fish retina during normal development. One intriguing exception to this is the regeneration of UV cones during the natural ontogeny of adult salmonid fish, replacing UV cones that are lost during development of juvenile fish [8]. This loss of UV cones from the retina is associated with times that salmonids go to deeper and/or marine waters. The loss of UV cones appears to permit or instruct the addition of new rod photoreceptors into the spaces created, presumably to facilitate new visual tasks in the lower light environs [8], [43], [44]. Return of the fish to shallow spawning grounds, in many populations representing a long distance migration and return to freshwater, may be associated with regeneration of the UV cone [65]. This natural regeneration of cones in salmonids is the exception, however, and most retinal regeneration research has focused on a variety of cell damage paradigms that induce regeneration of many cell types [13]. In sum, regeneration of cones is the primary focus of clinical interest but typically represents only a small portion of the complex regeneration processes studied in available models.

The natural regeneration of UV cones during salmonid ontogeny, inducible by alterations in thyroid hormone signalling, was previously advanced [8] as a tractable model to examine cone regeneration because of three distinct advantages: i) only a single cone class is regenerating, reducing the complexity of molecular and cellular events compared to regenerating many cell types; ii) the timing of events could be controlled; iii) the simplicity of the natural cone loss means that the tissue that cells are regenerating into is intact, unlike the unknowably random damage to tissue one envisages following toxic lesions. As an overall aim, the current work seeks to combine these advantages from trout with the power of zebrafish genetics and biomedical modeling so we can better understand how cone photoreceptors are regenerated and rewired into the neural retina.

We established a chemically-inducible cone ablation method (ablating UV cones, homologs of UV cones in salmonid fish and of the “S cones” or “blue cones” in humans) and demonstrated that the identity of the regenerating cells matches the identity of the ablated cell type. Thus our work points to the existence of unidentified mechanisms that bias stem cells to produce the same photoreceptor subtype that was ablated. This remarkable mechanism is worthy of detailed study because it can inform vision science researchers as they develop stem cell therapies and induce proliferation of Müller glial cells to repair vision loss; such studies must learn how to generate cone photoreceptors to repair precious daytime vision. Though the progress in murine models showing regeneration of rods is very promising [19], [24]–[27], including recent demonstrations showing restoration of visual function, replacement of cones [19]–[23] will be challenging to model in nocturnal murine animal models that possess few cones. Expansion of the current regeneration paradigm into adult fish retina will be an important step in uncovering mechanisms that inform development of therapeutics.

### A chemically-inducible model of cone photoreceptor ablation and regeneration

Our goal was to express a transgene in the majority of UV cones in the adult fish retina to enable prodrug-induced ablation, such that we could analyze the identity of cells appearing into the UV cone position of the cone mosaic. This would allow us to make the simplifying assumption that the cells reappearing into the UV cone position of the mosaic were regenerated. The novel model we report here does not fully accomplish this, as not every UV cone expresses the required transgene and this shortcoming is exasperated later in development. We overcame this difficulty in part by identifying regenerating cells via their incorporation of BrdU.

Further, we have not formally proven that other cell types are never ablated by our treatments, though we saw no evidence of such events and Figures 4 and 5 provide evidence that they are rare. Similarly, we have not ruled out that other cell types, such as adjacent cones, might not subsequently die in the days or weeks following UV cone ablation. These potential caveats should be borne in mind when interpreting the response to cone ablation. However, when placed in the context of past methods used to ablate cone photoreceptors (e.g. surgery, bright light or neurotoxins, see Introduction and ref [13]), it is imminently reasonable to describe this ablation method as ‘specific’ in regards to the preferential ablation of UV cones.

Following MTZ-induced ablation of the transgene-expressing UV cones, a significant increase in proliferation was observed in both the INL and ONL of the retina. The proliferative response was detected 24 hours after MTZ treatment, and was evident months later, providing evidence that the ablation of a subset of photoreceptors is sufficient to stimulate proliferation and retinal regeneration. It is accepted that Müller glial cells are the major source of neuronal proliferation in the INL of developed retina, responding to retinal injury [57], [58] manifested here as UV cone ablation (though we cannot exclude a contribution of regenerate from the CMZ in our model). The appearance of immature retinal neurons was observed at 5 days post MTZ treatment as evidenced by the incorporation of BrdU in the nuclei of cells located in the ONL. Various BrdU-positive cells co-localized with NTR-mCherry transgene expression at this time point, indicating that at least some of these precursors were expressing the UV opsin gene and had already been specified to a particular photoreceptor fate. The regeneration of morphologically typical mature cone photoreceptors expressing UV-sensitive opsin was observed at 1 week following ablation, confirming the value of the MTZ-NTR ablation method for neuronal regenerative investigations. This conditional targeted ablation technique is an efficient and effective method for removal of a particular cone photoreceptor population, providing both temporal and cell-type specificity.

### Cone photoreceptors are added to mature zebrafish retina during normal development

As discussed above, continual addition of rod photoreceptors to the central mature retina occurs throughout the life of most fish, but addition of cone photoreceptors is only known to occur in select species [8]. Thus we were surprised to consistently identify approximately 20 cone photoreceptors that had recently been proliferating (BrdU+) in retinas that had received no induction of damage. The area surrounding the optic nerve head, where we observed these BrdU+ cone photoreceptors, is the first piece of retina generated during fish development, and thus is denoted as a “larval remnant” [31]. This larval remnant can be observed *in vivo*, and is thus does not represent an artefact of the dissection process [66]. Past observations, including unanticipated variability in the relative abundances of cone types between individuals and between ontogenetic stages, led to the hypothesis that the larval remnant might be remodeled during ontogeny [31]. It is noteworthy that the larval remnant retains a heterotypic arrangement of cone mosaic, but the arrangement is not organized into rows like the later-generated retina [31]. Our data confirms that the larval mosaic is indeed remodeled, at least through addition of new cone photoreceptors. It is possible that this remodeling within the larval remnant has adaptive value for the fish. On the other hand, we suggest it is driven by continual disruption of this region by expansion of the adjacent optic nerve head that must occur to accommodate the addition of ganglion cells at the retinal periphery, and the addition of new optic nerve axons through this region [67]. Thus addition of new cone photoreceptors occurs during natural development of fish in taxa beyond salmonids, and although it occurs only in a narrow and particular context, this observation is important to consider during interpretation of experiments that affect retinal degeneration and regeneration in fish.

### Loss of UV cones does not induce generation of rod photoreceptors in their place

In rainbow trout, the loss of UV cones has been noted to be coincident with an increase in rod photoreceptors [8], [43], [44]. Indeed an augmentation of visual abilities by adding rods in concert with migration to deeper waters could be the causal adaptive value that leads to loss of UV cones during salmonid ontogeny. Changes observed in the abundance of opsin and chromophore types during these transitions [8], [43], [44], [68]–[70] are consistent with this type of remodeling being associated with new visual tasks. Regardless of ultimate mechanisms, the data from trout and salmon led us to predict that rod photoreceptors would take the place of lost UV cones in our zebrafish model. We refuted this hypothesis, as there was a significant decrease in the relative abundance of rods generated following UV cone ablation. Thus loss of UV cones is not itself a simple conserved trigger for generating new rods into the retina.

### Ablation of UV cones leads to regeneration of cones

We found that when ablation was mostly limited to UV cones, it led to a significant increase in the number of proliferating cells that became cones. Regarding the subtype identity of these regenerated cones, future work is required to resolve whether any bias exists towards replacing the cognate cone subtype. Our data do not eliminate this possibility, as indicted by the observation that 30.1% of BrdU-positive cones were UV cones following UV cone ablation. This is higher than the expected proportion, because UV cones compose 16.7% of the cones in normal retina (χ^2^ p = 0.0002, n = 311 BrdU+ cones in six fish). This increase contrasts the observations in control retinas, where 18.3% of the BrdU+ cones were UV cones (χ^2^ p = 0.769, n = 84 BrdU+ cones in six fish), validating 16.7% as a reasonable expected value. However the interpretive power of this result is tempered by inter-individual variability amongst the small number of adult fish that passed our criteria for inclusion in analyses (see Methods, n = 6 treated fish, n = 6 vehicle control fish evenly split between two trials); Indeed non-parametric statistical tests fail to reveal a difference in the proportion of UV cones regenerated when calculated as a mean per individual animal (p = 0.064). Importantly, there was also an increase in the number of proliferating cells that became cone subtypes other than UV cones (as a proportion of all BrdU-positive photoreceptors). This increase to the sum of the other three cone subtypes was smaller, and also did not reach statistical significance (p = 0.075). Thus future work will be required to determine if there is a bias of cone regeneration towards replacing the ablated cone subtype. This should include increasing sample sizes, both regarding number of fish and number of cones ablated, to assess inter-individual variability. It will also be instructive to quantify additional cone subtypes to determine which are reduced in relative abundance coordinated with the hypothetical increase in UV cone abundance, if any. Such experiments should also test the assumption that specific ablation of a particular cone subtype does not subsequently lead to death of other photoreceptors, as this may impinge upon what cells would be expected to regenerate. Furthermore, the conclusion will ultimately depend upon comparison to animal models that ablate other cone subtypes, to assess whether regeneration is a concerted response coordinated to replace the ablated cone subtype.

Because cones regenerate following cone ablation, we conclude that a mechanism must exist whereby regenerating cells are somehow instructed to have or acquire the correct identity. We envisage a pluripotent progenitor pool is activated upon UV cone ablation, and non-cell-autonomous mechanisms instruct the specification of the regenerating cells. We note that ‘rod progenitors’, apparently becoming more abundant with ablation in our study, may be especially able to generate UV cones. In this regard it is interesting to consider evidence for a common pathway leading to rods and UV cones, suggested by mutation of the transcription factor *Tbx2b* leading to differentiation of rods in place of UV cones in zebrafish [71]. Thus we interpret our data to mean that ‘rod progenitors’ that would otherwise be giving rise to rods during normal development have been redirected in our paradigm to regenerate cones.

### Building heterotypic cell mosaics

From the existence and precision of the zebrafish cone mosaic, one can infer that information about neighbour identity is integrated into mechanisms directing the differentiation or movement of newly generated cones (Fig. 1). Such explanations are especially attractive when modelling the addition of new cones at the retinal periphery, as it appears to be building upon the existing mosaic as a template (see right side of Figure 1). From a wealth of data interrogating intercellular communication mechanisms leading to heterotypic spatial arrangements of *Drosophila* photoreceptor spacing, lateral induction mechanisms have been anticipated as causal in creating fish cone mosaics [29], [31], [34], [72]. Such hypotheses correlate adequately with the temporal dynamics of cone differentiation at the retinal margin [29], [42]. However these mechanisms are not formally required to construct a heterotypic cell mosaic [73]–[75], and movement of cells can be sufficient [75]. Indeed mathematical modelling of fish cone mosaic formation can recreate the cone mosaics observed in fish by permitting movement of fully differentiated cells, under the auspices of differential adhesion between cell types [76]–[78]. For example, the UV-cone marked ⌘ in Figure 1 could differentiate into the incorrect position, and subsequently move via adhesive or repulsive mechanisms. We are not aware of previous experimental interventions that have demonstrated that the specification of a vertebrate cone is indeed influenced by the identity of its neighbors. Our conclusion that ablation of UV cones leads to regeneration of cones, regardless of any hypothetical bias towards generating UV cones (see section immediately above), leads us to favor the proposal that mosaic-building mechanisms involve non-cell-autonomous induction of cell fate (consistent with the * and ☠ in Fig. 1). Alternatively, our data are also consistent with the ablated UV cone releasing a signal that directs the fate of nearby stem cells; however this concept is challenging to integrate into the concept of cone addition templated by the existing mosaic during expansion at the CMZ. In sum, cell movements are sufficient to build this heterotypic cell mosaic in mathematical models, and may also occur, but our data argue that induction of cell fates from neighboring cones is occurring *in vivo*. This data confirms that the identity of regenerating cones is not random, and provides evidence that the specification of a differentiating cone (and not just its position) is influenced by the identity of its neighboring cells. This contrasts observations of cone mosaics following ablation of many cone photoreceptors, where normal neighbor relationships and cell densities were not regenerated [1], [36]. This difference is likely the result of the regenerating cones integrating more complex and variable signals following a less precise retinal damage paradigm.

### Relevance to mammalian photoreceptor regeneration

Recent work suggests two routes of treating retinal degenerative diseases through regeneration. One intriguing possibility is the activation of Müller glia in mammals to a stem cell function, inspired by the role of Müller glia in fish [58], and ongoing work supports that this approach has promise [79]–[81]. An alternative is transplantation of stem cells or progenitor cells into diseased retinas. The latter includes isolation of progenitors (as they differentiate into nascent photoreceptors) based on their expression of transcription factors, coupled with transplantation into mouse models of retinal degeneration. This approach shows promise, as rod photoreceptors can be generated [19], [24]–[27], and have recently been demonstrated to replace visual function [25]. Strikingly, though, parallel efforts to replace lost cone photoreceptors by several groups are not meeting with the same success [19]–[23]. Considering the desirability of restoring daytime vision, this is an important hurdle for clinically relevant stem cell therapies to clear. Some hope emanates from the speculation that the cone-poor, rod-rich murine retina is a poor approximation of the cone-rich human fovea; Thus it may be that one factor limiting the success of approaches designed to replace lost cones is their destination, and cone-rich retinal regions might support successful cone regeneration. Our data demonstrating regeneration of cones, and a decrease in the proportion of rods, are not inconsistent with this notion. It is noteworthy that the current work was performed in young fish, due to the transgene expression becoming less tractable at later stages, yet the regeneration quantified here was in post-developmental retina, after formation of functional vision. Expansion of our paradigm into adult fish will set the stage to discover intercellular mechanisms of cone specification, some of which may well be impactful in design of cell-based treatments with a goal of functionally replenishing the human fovea.

### Conclusion

We developed a chemically inducible cone photoreceptor ablation paradigm that has allowed a unique view of photoreceptor specification during regeneration and development. We anticipate this will enable future studies wherein a reductionist approach can interrogate cone regeneration processes in the context of an intact *in vivo* system.

Our data suggest that the specification of a photoreceptor is dependent on the neighboring photoreceptors’ identity, though other interpretations were discussed. Thus, ablation of a particular cone subtype led primarily to regeneration of cones, and the existence of mechanisms driving this developmental process can now be explored. It remains to be seen if similar mechanisms operate robustly throughout the CNS, and throughout diverse taxa, or if the precision of the heterotypic cell arrangements in the retina of fish is required to direct this specification. Regardless, such mechanisms may prove influential in design of retinal repair therapies where one hopes to direct stem cell fates towards integration of cone photoreceptors into the cone-rich fovea and thereby repair daytime vision.

## Methods

### Zebrafish maintenance

Zebrafish (*Danio rerio*) were raised using standard procedures [82]. Some larvae were treated with PTU (1-phenol-2-thiourea) to block formation of melanin pigment. Fish were maintained at 28°C under standard fluorescent lights and were fed twice daily with brine shrimp or flaked food.

### Ethics Statement

All protocols were approved by the Animal Care and Use Committee: Biosciences at the University of Alberta as dictated by the Canadian Council on Animal Care.

### Constructs for transgenesis

We engineered transgenic zebrafish to express the *E. coli* gene *nfsB* encoding the nitroreductase (NTR) enzyme fused to a fluorescent marker gene, mCherry, to allow for visualization. In this method, NTR is driven by a cone-specific opsin promoter, resulting in its expression in the targeted neuron type (UV cones). These cones survive normally until the application of a prodrug, metronidazole (MTZ), which binds to NTR and is electrochemically reduced, converting it into a DNA cross-linking cytotoxin [51]. This results in precise cell ablation of the subset of cones expressing NTR. Ablation is terminated by removing fish from the MTZ treatment, allowing for regeneration of the ablated cones to occur and resulting in the restoration of fluorescence (Fig. 2) [51].

We engineered zebrafish that express NTR (*nfsb*) fused to mCherry in UV cone photoreceptors. We generated zebrafish that express the transcriptional activator Gal4-VP16 under control of the SWS1 opsin promoter and bred these to zebrafish expressing NTR-mCherry under control of the UAS promoter. Constructs for transgenesis were generated using multisite Gateway cloning into vectors amenable to Tol2 recombination [83]. A cone photoreceptor-specific promoter was used to create the p5E-*sws1* plasmid, to direct expression in UV- sensitive cones. This promoter is equivalent to that published previously [49], denoted *-5.5opn1sw1* (ZDB-GENE-991109-25; previous nomenclature zfSWS1-5.5A). Gateway plasmid p5E-*sws1* was created by subcloning 5.5 Kb of the SWS1 opsin promoter from a plasmid, kindly provided by Shoji Kawamura [49], into p5E-MCS using *Sal*I and *Xho*I restriction enzymes. This plasmid was then combined with pME-Gal4VP16, p3E-polyA and pDestTol2CG2 (generously provided by Chi-Bin Chien) via an LR reaction [83] to generate the construct pDestTol2CG2;*sws1:*Gal4VP16-polyA. This construct is expected to drive expression of Gal4-VP16 in UV cones, and also express GFP in the heart muscle to facilitate identification of transgenic individuals. The final construct was validated by sequencing (not shown).

### Generating and characterizing transgenic zebrafish

The construct above was used to generate stable transgenic zebrafish by injecting it (750 ng) with Tol2 mRNA (25–30 pg) generated as previously described [83] into hemizygous *Tg(-5.5opn1sw1:EGFP)kj9* zebrafish (ZDB-GENO-080227-6) expressing GFP in UV cones. These fish were raised if appropriate and screened for stable transgenesis by examining the F1 generation (outcross to UAS:*nfsb*-mCherry for Gal4-VP16 transgenics) for mCherry expression in cone photoreceptors.

### Optimizing metronidazole-nitroreductase ablation

Transgenic zebrafish [*Tg(SWS1:Gal4-VP16)ua3016;Tg(UAS-E1b:NfsB-mCherry)c264*] were placed in a metronidazole (Sigma-Aldrich, St. Louis, MO; No. M3761) solution with E3 media or system water to induce MTZ-NTR ablation. To optimize the treatment, various MTZ doses were tested (1 mM, 2.5 mM, 5 mM, 6 mM, 6.5 mM, 7.5 mM and 10 mM), with and without DMSO, and incubation time ranged from 24–60 hours. The resulting optimized treatment, 10 mM MTZ solution in 0.2% DMSO for 48 hours at 28°C, was applied to all subsequent ablation experiments with larvae, with treatment beginning at 4–6 days post fertilization (dpf). Following treatment, larvae recovered in E3 media for 8–24 hours, with at least 2 changes of fresh E3 to wash away remaining MTZ [51].

### Preparing cryosections

Frozen retinal sections were prepared as described [8]. Larvae were placed in fixative (4% paraformaldehyde/5% sucrose/0.1 M phosphate buffer pH 7.4) overnight at 4°C, and were embedded in Tissue-Tek O.C.T. embedding compound (Sakura Finetek; No. 4583). 10 µm-thick cryosections were thaw mounted to SuperFrost Plus glass slides (Fisher, Pittsburgh, PA; No. 12-550-15). Sections were air dried at room temperature for 30 minutes and stored at −80°C until use.

### Immunohistochemistry on retinal sections

Sections were air-dried upon removal from the −80°C freezer and blocked for at least 30 minutes at room temperature using 10% normal goat serum in phosphate buffer saline, pH 7.4 (PBS) with 1% Tween-20. The sections were incubated in a humid chamber with primary antibody diluted with 2% normal goat serum in PBS/1%Tween-20 overnight at 4°C. Primary antibodies included anti-BrdU rat 1∶20 [Accurate Chemical, Westbury, NY; No. OBT0030S], anti-BrdU mouse 1∶50 [BD Pharmingen, San Diego, CA; No. 555627]; anti-DSRed (mCherry) mouse monoclonal 1∶200 [Clontech, Mountain View, CA, No. 632393], anti-GFAP 1∶100 [zrf-1 from ZIRC, Eugene, OR, ZDB-ATB-081002-46], anti-Glutamine synthetase 1∶50 [Millipore, MAB302, Zfin ID ZDB-ATB-081009-5], and/or anti-zpr1 mouse monoclonal 1∶200 [ZIRC, ZDB-ATB-081002-43]. Tissue was rinsed twice and washed in 1% normal goat serum in PBS or in PBS/1% Tween-20 for at least 30 minutes at room temperature. Primary antibodies were detected by incubating sections in a humid chamber with secondary antibodies anti-mouse or anti-rat conjugated to AlexaFluor fluorochromes 350, 488, 555 or 647 (Invitrogen, Carlsbad, CA), diluted 1∶1000 in 2% normal goat serum in PBS/1% Tween-20, at room temperature for at least 1 hour or overnight at 4°C. Tissue was rinsed and washed in 1% normal goat serum in PBS/1% Tween-20 following secondary antibody exposure. Nuclei were detected with 1 mg/L DAPI (incubated for 1–2 mins; Invitrogen, Carlsbad, CA), 1∶5000 of TO-PRO-3 (incubated for at least 10 minutes, Invitrogen No T3606) or 2 µg/ml Hoechst 34580 (incubated for 10–15 mins; Invitrogen, Carlsbad, CA, No H21486) diluted in PBS.

### BrdU application to fish and antigen retrieval on sections or whole mount retinas

Larvae were incubated in 5 mM BrdU (5-bromo-2'-deoxyuridine; Sigma-Aldrich, St. Louis, MO; No. B5002) dissolved in E3 for 24–48 hours after recovery from MTZ. For quantifying proliferation, larvae also received BrdU treatment for 24 hours prior to MTZ treatment. Larvae were maintained and fed fry powder until they were selected for analysis.

Tissue (cryosections or whole mount neural retinas) was incubated in 2N HCl in PBS/1% Tween-20 for 30 minutes at room temperature to expose the BrdU antigens. Tissue was washed in PBS/1% Tween-20 for 15 minutes at room temperature, then in PBS for 15 minutes at room temperature. The immunohistochemistry procedure (described above), beginning with blocking, was initiated at this point.

### In situ hybridization and immunocytochemistry of whole retinas

To label UV cones, a fluorescein-labelled riboprobe was prepared against short wavelength sensitive 1 opsin (*sws1*), synthesized as previously described [31]. To label all blue, green and red cones of the retina, a cocktail of digoxigenin-labelled riboprobes against the short wavelength sensitive 2 opsin (*opn1sw2*, accession No. AF109372, ZDB-GENE-990604-40), the four medium wavelength sensitive opsins (*opn1mw1*, *opn1mw2*, *opn1mw3*, and *opn1mw4*, accession Nos. AF109369, AB087806, AB087807, and AF109370, ZFin ID: ZDB-GENE-990604-42, ZDB-GENE-030728-5, ZDB-GENE-030728-6, and ZDBGENE-990604-43, respectively) and the two long wavelength sensitive opsins (*opn1lw1* and *opn1lw2*, accession Nos. AF109371 and AB087804, ZDB-GENE-990604-41 and ZDB-GENE-040718-141, respectively) were used. Full-length antisense riboprobes (varying in length; see accession Nos.) were synthesized from linearized plasmid in each case. All 8 riboprobes were mixed into a cocktail and applied in excess to isolated adult retinas as described previously [31], except hybridization temperatures and post-hybridization washes were at 65°C. Riboprobes were detected in sequence using anti-fluorescein (antibody produced by direct immunization of fluoroscein into sheep; then, ion-exchange chromatography and immunoabsorption were used to isolate IgG; Roche Diagnostics; No. 11426346910) then anti-digoxigenin (antibody produced by direct immunization of digoxigenin into sheep; then, ion-exchange chromatography and immunoabsorption were used to isolate IgG; Roche Diagnostics; No. 11207733910) antibodies conjugated to peroxidise (POD). After application of the antibody as previously published [31], the tissue was incubated in tyramide-conjugated to AlexaFluor 488 or 555 (Invitrogen, Carlsbad, CA; Nos. T20932 and T30955) as per the manufacturer’s protocols. After development of each fluorescent signal, the antibody was deactivated by incubating the tissue in 1.5% H_2_O_2_ for 30 minutes at room temperature. After several washes with PBS/1% Tween-20, the tissue was probed with the next antibody and the appropriate tyramide-conjugated fluorochrome.

Following *in situ* hybridization and visualization of all signals, BrdU was retrieved and detected with rat anti-BrdU antibody by immunohistochemistry methods described above except that the blocking solution and antibody dilutions were made with normal goat serum diluted in PBS/BSA/DMSO/Triton X-100.

### Quantification of ablation from retinal sections and assay of cell death

*Tg(SWS1:Gal4-VP16)ua3016;Tg(UAS-E1b:NfsB-mCherry)c264* fish, treated with either a MTZ solution or a control DMSO solution, were quantified for conditional ablation of UV cones. Employing cryosections of retinal tissue, a representative section for each larval fish was examined. A ratio of the number of UV cones expressing mCherry per length of ONL ( µm) was calculated for each retina, and the average was taken for each treatment group. Samples were acquired from fish fixed after a treatment of 10 mM MTZ (or DMSO) for 48 hours (n = 10 vehicle-only control fish, n = 8 experimental fish receiving MTZ).

To quantify the proportion of total UV cones that are ablated in transgenic fish, *Tg(SWS1:Gal4-VP16)ua3016;Tg(UAS-E1b:NfsB-mCherry)c264* larvae were screened for GFP and mCherry expression in the UV cones, following treatment with either 10 mM MTZ or vehicle control, fixed 2 days post-treatment. Retinas were dissected away from other ocular tissues, flatmounted as whole retina and imaged on a Zeiss LSM 700 confocal microscope mounted on a Zeiss Axio Observer.Z1 and using ZEN 2010 software (version 6.0, Carl Zeiss MicroImaging). UV cones expressing either GFP, mCherry, or both were counted using ImageJ64 for Mac (Wayne Rasband, National Institutes of Health, USA; http://imagej.nih.gov/ij).

TUNEL detection was performed to identify apoptotic cells on retinal cryosections (Roche, Laval, QC; No. 11684817910). Retinal tissue sections on slides were incubated in blocking solution (3% H_2_O_2_ in methanol) for 10 minutes at room temperature, then rinsed with PBS. Slides were next incubated in fresh permeabilization solution (0.1 M sodium citrate/0.1% Triton X-100) for 2 minutes at 4°C, then rinsed with PBS. Label solution and Enzyme solution were combined to form the TUNEL reaction mixture and applied to slides as per the manufacturer’s protocol for cryopreserved tissue. After a 60 minute incubation in a humid chamber at 37°C, slides were rinsed with PBS and analyzed for fluorescence. The green signal was converted if tissues were already expressing GFP in the UV cones via Converter-POD (supplied in the kit) which was applied to sections for 30 minutes at 37°C in a humid chamber followed by tyramide conjugated to AlexaFluor 647 (Invitrogen, Carlsbad, CA; No. T20936) application as described above. To quantify total cell death, TUNEL-positive cells were quantified per section on one representative section per fish. To calculate the abundance of dying cells that were and were not UV cones, a confocal stack encompassing the entire area of each retinal section was collected through the depth all TUNEL+ elements. These files were examined to identify each TUNEL+ signal as either mCherry positive (indicating it was a UV cone) or not, using Imaris software, and results were tallied.

### Quantification of proliferating cells from retinal sections

Larval *Tg(SWS1:Gal4-VP16)ua3016;Tg(UAS-E1b:NfsB-mCherry)c264* zebrafish were treated with 5 mM BrdU for 24 hours at 4 dpf, incubated in 10 mM MTZ (or DMSO) for 48 hrs from 5–7 dpf and then were exposed to 5 mM BrdU again for up to 48 hours. Sample fish were fixed at 24 hours post MTZ treatment (control n = 9, experimental n = 10). Cryosections of retinal tissue were analyzed for BrdU incorporation into cells in the INL and ONL. A representative section from each eye of an individual fish was quantified whenever possible and the average was taken. Quantification of proliferating cells in the INL and ONL was performed by measuring the area ( µm^2^) of the retina and counting the number of BrdU-positive cells within the retina, excluding those in the CMZ. A ratio of the number of BrdU-positive cells in the INL or ONL per area of retina ( µm^2^) was calculated for each fish. To quantify the size of the CMZ, the area of the CMZ relative to the area of the retina was measured using ImageJ64 for Mac. Values are reported as ratios of the CMZ area to total retinal area ±SE.

### Analyzing whole mount retinas to determine the identity of BrdU-positive photoreceptors

Juvenile *Tg(SWS1:Gal4-VP16)ua3016;Tg(UAS-E1b:NfsB-mCherry)c264* zebrafish were subjected to a series of BrdU and MTZ (or DMSO) treatments, dissolved in system water, over the course of 1 week (7 dpf: 24 hours in 5 mM BrdU; 8 dpf: 24 hours in 10 mM MTZ [or DMSO], 9–10 dpf: 48 hours in system water, 11 dpf: 24 hours in 10 mM MTZ [or DMSO], 12 dpf: 24 hours in system water, 13–14 dpf: 48 hours in 5 mM BrdU). Fish were fed twice daily. Following recovery from treatments, fish were placed in the aquaria system to grow.

At either 3 or 5 months post-fertilization, 10 fish treated with MTZ and 10 fish treated with DMSO vehicle were dark-adapted overnight to prepare for retinal dissection. The fish were deeply anesthetized with MS-222 and euthanized by severing the brain stem. The eyes were removed from the head and the retinas were carefully dissected and separated from the retinal pigment epithelium (RPE). The whole retinas were placed into fixative (4% paraformaldehyde/5% sucrose/0.1 M phosphate buffer pH 7.4) overnight at 4°C. Following *in situ* hybridization and BrdU detection (as described above), only retinas that had the region of the optic nerve intact were chosen to flat-mount on glass slides (MTZ treated n = 7; DMSO treated n = 7).

The larval remnants of the whole mount retinas, proximal to the optic nerve, were examined for BrdU incorporation into photoreceptors via confocal microscopy. For each retina, 2 rings of BrdU-containing cells were visualized, representing the cells that had been proliferating in the CMZ at each time of BrdU application. The innermost ring represented the earliest BrdU treatment at 7 dpf for 24 hours, while the outer ring represented the second BrdU treatment at 13 dpf for 48 hours. Analysis of the larval remnants excluded the cells that contributed to either ring. Only BrdU-positive cells that were clearly distinguishable as in between the 7- and 13-day rings or within the 7-day ring were included (Fig. 8).

A series of z-stack confocal images of portions of each larval remnant were taken for analysis of BrdU co-localization with photoreceptor subtypes such that the researcher was blinded to the treatment. As expected, BrdU incorporation in the nucleus of a photoreceptor was typically positioned vitreal to the *in situ* hybridization labelling of the photoreceptor opsin. Therefore analysis was performed using the ZEN software (2009; Carl Zeiss MicroImaging), employing the Ortho tool which allows for 3-dimensional analysis of a z-stack, enabling the visualization of opsin and BrdU within the same cell (Fig. 8B-D, S2). BrdU-positive cells in the scleral portion of the ONL that did not co-localize with a cone photoreceptor opsin were counted as rods or rod progenitors but herein we make the simplifying and conservative assumption that they were rods. The BrdU-containing cells were classified into six categories based on opsin *in situ* hybridization and nuclear position: i) cells that were unambiguously UV cones, ii) cells that were unambiguously BGR (blue, green or red) cones, iii) cells that were unambiguously rods, iv) cells that could not be distinguished, being possibly UV or BGR cones, v) cells that were possibly UV cones or rods, and vi) cells that were possibly BGR cones or rods. UV and BGR cones were considered identified unambiguously if their nucleus was contiguous with only one opsin riboprobe signal. Following classification, only the unambiguous categories were considered for further analysis (i, ii and iii) because the ambiguous categories included only a small percentage of total cells and the distribution was not significantly different amongst treatments (maybe UV or BGR p = 0.555; maybe UV or rod p = 0.13; maybe BGR or rod p = 0.389).

Following quantification of BrdU-incorporated cells and analysis of photoreceptor identity, some retinas were excluded from the remainder of the analysis based on the quantity of BrdU-positive cells. Retinas with fewer than 20 BrdU-positive cells were excluded. The remaining viable retinas (MTZ treated n = 6; DMSO vehicle treated n = 6) were included. The percentage of BrdU-positive cells for each of the three categories (UV, BGR, and rods) following MTZ or DMSO vehicle control treatment was calculated for each retina, and the average was taken.

### Statistical analysis

Mann-Whitney U tests and Kruskal-Wallis Analysis of Variance tests with post-hoc Tukey's Honestly-Significant-Difference Test were performed using SYSTAT 12 (2007) software package to generate *t* values, degrees of freedom, standard error of difference, and p values. Sample sizes reported as n = number of fish examined. χ^2^ tests were performed in Excel (Microsoft). Standard error of the mean was calculated and is represented as error bars in the appropriate figures.

## Supporting Information

Figure S1 **Assessing the quality of transgene expression in photoreceptors.** We assessed our novel transgenic driver line, intended to express Gal4-VP16 in UV cone photoreceptors, by breeding it to two reporter lines. One reporter, primarily used in our experiments, drives expression of *nfsB-mCherry* (NTR-mCherry protein), and the other reporter is GFP. Thus breeding created *Tg(SWS1:Gal4-VP16)ua3016;Tg(UAS-E1b:NfsB-mCherry)c264*;*Tg(4xUAS:GFP)hzm3* zebrafish. The inset, from a fish at 22 days post-fertilization (dpf), is magnified at the bottom of the figure and is consistent with data from fish at 42 or 66 dpf, regarding GFP being present in more cones than mCherry. Thus we cannot rule out deficits with the Gal4-VP16 driver line as contributing to the lack of robust NTR-mCherry expression in all UV cones. Further, the quantity of *nfsb-mCherry* expressing cells is substantially decreased in older fish.
(TIF)

Figure S2 **Confocal z-stack analysis to determine the identity of BrdU-positive photoreceptors.** A three-dimensional analysis was performed using the ZEN microimaging software to allow for the visualization of BrdU in the nucleus co-localizing with opsin expression. Photoreceptors were divided into 3 unambiguous categories: BrdU-positive co-localizing with UV opsin (A), BrdU-positive co-localizing with BGR opsin (B), and non-colocalizing BrdU-positive rods (C). The BrdU+ rod in C is located in the vitreal side of the ONL compared to panels A and B, thus cells towards the right of the panel lack cone opsin labelling.
(TIF)

Movie S1 **The proliferating cells that increase in abundance during regeneration include Müller glia in the INL, as indicated by close apposition of Müller glia markers (green) with BrdU+ nuclei (magenta).** Example shown is a cocktail of two antibodies against Müller glia (green, anti-GFAP & anti-glutamine synthetase, both raised in mouse). Saturated green at top of figure is from UV cones expressing abundant GFP. See also Figure 6 G-I.
(MPG)

Movie S2 **The proliferating cells that increase in abundance during regeneration include Müller glia in the INL, as indicated by close apposition of Müller glia markers (green) with BrdU+ nuclei (magenta).** Example shown is with an antibody against Müller glia (green, anti-glutamine synthetase). Saturated green at top of figure is from UV cones expressing abundant GFP.
(MPG)

Movie S3 **Tangential view of the proliferating cells that increase in abundance during regeneration include Müller glia in the INL, as indicated by close apposition of Müller glia markers (green) with BrdU+ nuclei (magenta).** Example shown is a cocktail of two antibodies against Müller glia (green, anti-GFAP & anti-glutamine synthetase, both raised in mouse). See also Figure 6 J-K.
(MPG)
