# Supplementary figures and images for: Regeneration of Cone Photoreceptors when Cell Ablation Is Primarily Restricted to a Particular Cone Subtype
Source: PLoS One. 2013 Jan 30;8(1):e55410. doi: 10.1371/journal.pone.0055410 (PMC3559598; doi:10.1371/journal.pone.0055410)

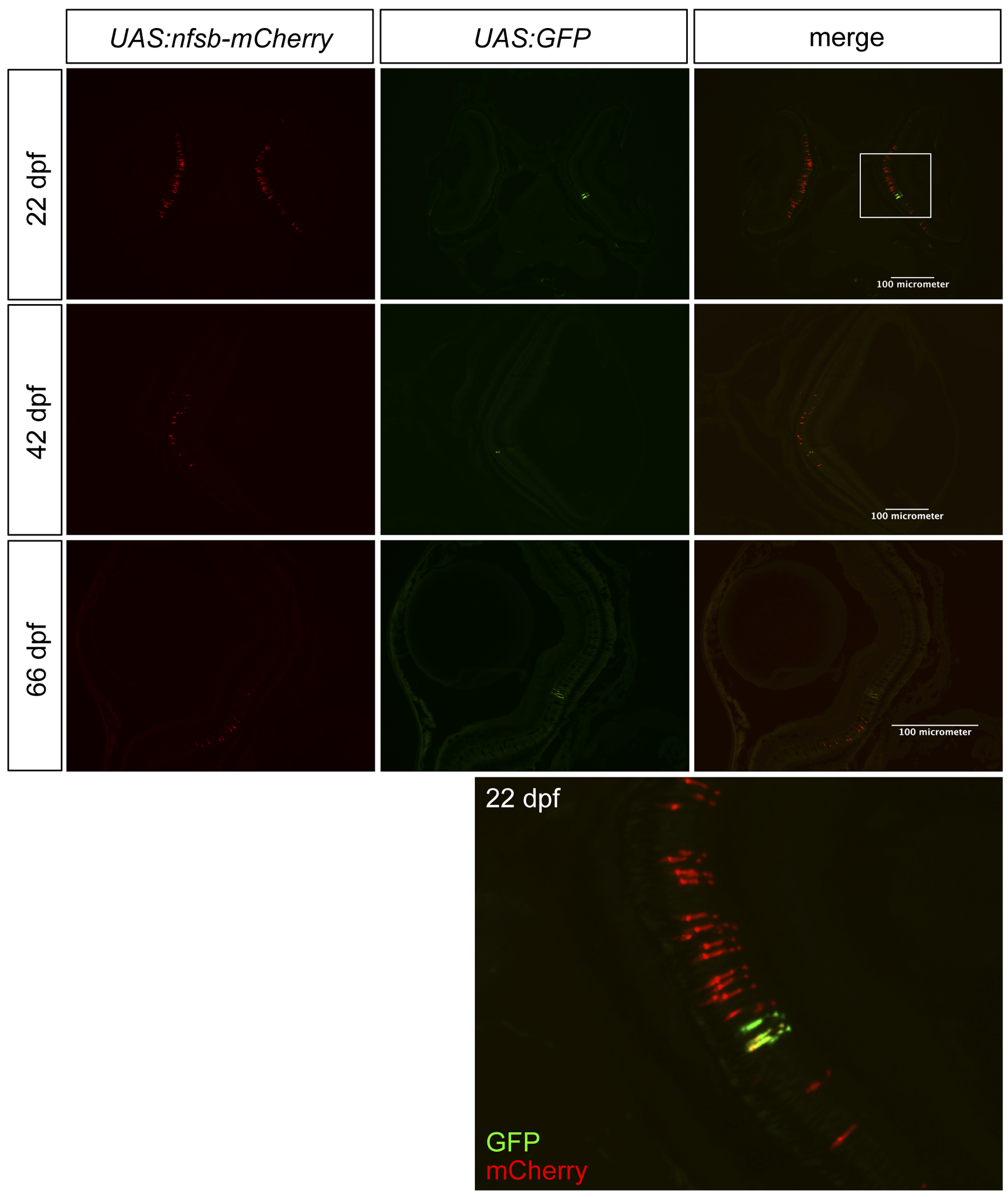

Supplement: Figure S1 — Assessing the quality of transgene expression in photoreceptors. We assessed our novel transgenic driver line, intended to express Gal4-VP16 in UV cone photoreceptors, by breeding it to two reporter lines. One reporter, primarily used in our experiments, drives expression of nfsB-mCherry (NTR-mCherry protein), and the other reporter is GFP. Thus breeding created Tg(SWS1:Gal4-VP16)ua3016;Tg(UAS-E1b:NfsB-mCherry)c264;Tg(4xUAS:GFP)hzm3 zebrafish. The inset, from a fish at 22 days post-fertilization (dpf), is magnified at the bottom of the figure and is consistent with data from fish at 42 or 66 dpf, regarding GFP being present in more cones than mCherry. Thus we cannot rule out deficits with the Gal4-VP16 driver line as contributing to the lack of robust NTR-mCherry expression in all UV cones. Further, the quantity of nfsb-mCherry expressing cells is substantially decreased in older fish. (TIF) [file pone.0055410.s001.tif]

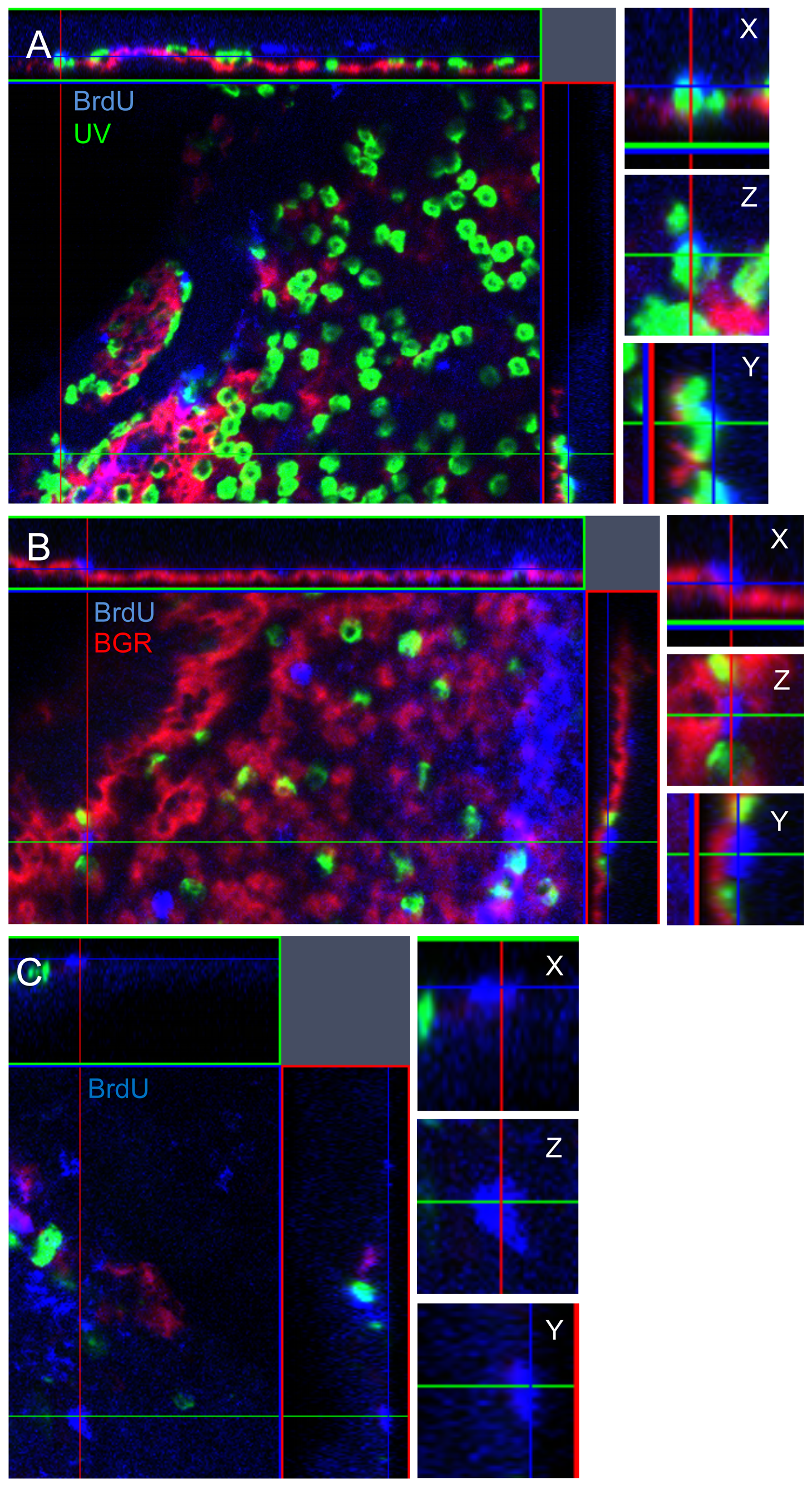

Supplement: Figure S2 — Confocal z-stack analysis to determine the identity of BrdU-positive photoreceptors. A three-dimensional analysis was performed using the ZEN microimaging software to allow for the visualization of BrdU in the nucleus co-localizing with opsin expression. Photoreceptors were divided into 3 unambiguous categories: BrdU-positive co-localizing with UV opsin (A), BrdU-positive co-localizing with BGR opsin (B), and non-colocalizing BrdU-positive rods (C). The BrdU+ rod in C is located in the vitreal side of the ONL compared to panels A and B, thus cells towards the right of the panel lack cone opsin labelling. (TIF) [file pone.0055410.s002.tif]
